# Characteristics of Poly(vinyl Alcohol) (PVA) Based Composites Integrated with Green Synthesized Al^3+^-Metal Complex: Structural, Optical, and Localized Density of State Analysis

**DOI:** 10.3390/polym13081316

**Published:** 2021-04-16

**Authors:** Shujahadeen B. Aziz, Muaffaq M. Nofal, Hewa O. Ghareeb, Elham M. A. Dannoun, Sarkawt A. Hussen, Jihad M. Hadi, Khayal K. Ahmed, Ahang M. Hussein

**Affiliations:** 1 Hameed Majid Advanced Polymeric Materials Research Lab., Physics Department, College of Science, University of Sulaimani, Qlyasan Street, Sulaimani 46001, Iraq; sarkawt.hussen@univsul.edu.iq (S.A.H.); khayal.ahmed@univsul.edu.iq (K.K.A.); ahang.hussein@univsul.edu.iq (A.M.H.); 2 Department of Civil Engineering, College of Engineering, Komar University of Science and Technology, Sulaimani 46001, Iraq; 3 Department of Mathematics and General Sciences, Prince Sultan University, P.O. Box 66833, Riyadh 11586, Saudi Arabia; muaffaqnofal@gmail.com; 4 Chemistry Department, College of Science, University of Sulaimani, Qlyasan Street, Sulaimani 46001, Iraq; hewa.ghareeb@univsul.edu.iq; 5 Associate Director of General Science Department, Woman Campus, Prince Sultan University, P.O. Box 66833, Riyadh 11586, Saudi Arabia; elhamdannoun1977@gmail.com; 6 Department of Medical Laboratory of Science, College of Health Sciences, University of Human Development, Sulaimani 46001, Iraq; jihad.chemist@gmail.com

**Keywords:** metal complex, coordination chemistry, UV-Vis, FTIR, XRD analysis, optical properties, bandgap study

## Abstract

The influence of dispersing Al-metal complex on the optical properties of PVA was investigated using UV–visible spectroscopy. Polymer composite films with various Al^3+^-complex amounts in the PVA matrix were arranged by solution casting technique by means of distilled water as a widespread solvent. The formation of Al^3+^-metal complex was verified through Ultraviolet–visible (UV-Vis) and Fourier-transform infrared spectroscopy (FTIR) examinations. The addition of Al-complex into the polymer matrix led to the recovery of the optical parameters such as dielectric constant (*ε_r_* and *ε_i_*) and refractive index (*n*). The variations of real and imaginary parts of complex dielectric constant as a function of photon wavelength were studied to calculate localized charge density values (N/m*), high-frequency dielectric constant, relaxation time, optical mobility, optical resistivity, and plasma angular frequency (*ω_p_*) of electrons. In proportion with Al^3+^-complex content, the N/m* values were amplified from 3.68 × 10^55^ kg^−1^ m^−3^ to 109 × 10^55^ kg^−1^ m^−3^. The study of optical parameters may find applications within optical instrument manufacturing. The optical band gap was determined from Tauc’s equation, and the type of electronic transition was specified. A remarkable drop in the optical band gap was observed. The dispersion of static refractive index (*n_o_*) of the prepared composites was analyzed using the theoretical Wemple–DiDomenico single oscillator model. The average oscillator energy (*E_o_*) and oscillator dispersion energy (*E_d_*) parameters were estimated.

## 1. Introduction

Polymer composites have been reported to be used as passive or active optical components for optoelectronics. Depending on their optical characteristics, they can be used as films with a high index of refraction, thin fififilm transistor, solar cells, light-emitting diodes, optical waveguides comprising materials, and photochromic materials [1]. The search for low-cost photovoltaic materials with an energy-efficient manufacturing process is becoming increasingly important [2]. The intensive and extensive survey of the literature confirmed that transition metals and semiconductor sulfides possess a narrow partially-filled band, which is well-separated in between the valence and conduction bands [3].

Precipitation, coagulation, electrochemistry, ion-exchange, and membrane-based technologies are standard techniques for extracting elements from such sources. However, all these methods suffer from some limitations, such as the generation of sludges from the high demand for chemical additives, high energy consumption, expensiveness, and low sensitivity [4]. Nowadays, major environmental pollutants are those heavy metals [5].

Recent developments in removing and recovering toxic substances have suggested biosorption as an innovative environmental remediation method [4]. The mass transfer process, known as sorption, involves converting a substance from the liquid phase to a solid surface, with the substance being bound by physical and/or chemical interactions. Sorption can serve as an inexpensive alternative to conventional processes due to the expansive surface area, high sorption capacity, and sorbents’ surface reactivity [5]. The economic and sustainable reasons for implementing biosorption as a bioremediation and resource management method are related to biomass use, such as plant extracts and microorganisms, like bacteria, fungi, algae, and yeast [6].

Both biosorption and adsorption have been widely proposed and employed as potential alternatives to the traditional chemical precipitation to eliminate heavy metals from wastewater at the wastewater treatment plants [7].

A different bio-remediation method for producing metal complexes was examined in the current study, including green remediation to separate the cations from transition metal salts. The past few years have increasingly seen the fabrication of metal nanoparticles by extracting plant leaves where silver and gold nanoparticles have been formed due to the reduction of their respective ions [8]. The reason for this is the existence of redox potential of the polyphenols in the extract from +0.09 to +0.4 V (redox potentials of Ag^+^/Ag and Au^+^/Au are +0.8 and +0.9 V, respectively) [9]. The significant role of conjugated double bonds of phenolic compounds in the quince leaf extract solution in reducing silver ions to silver nanoparticles was reported by Aziz et al. [10]. Since the redox potential of Cd^2+^/Cd is −0.40 V [11], another study found that the extracted tea plant polyphenols cannot reduce Cd^2+^ ions to Cd nanoparticles. However, the reaction of Al^3+^ ions with polyphenols in such systems leads to the formation of the Al^3+^-polyphenol complex. Wang revealed the capability of chelation between Fe^2+^ ions and polyphenols extracted from eucalyptus leaves while the polyphenols cannot reduce Fe^2+^ ion to Fe nanoparticles [12,13]. This is because the redox potential of Fe^2+^/Fe is −0.44 V. In another work, Wang et al. have utilized sage leaf extracts (Salvia officinalis) from Fe^2+^–polyphenols complexes [12,13]. However, they found that the extracted polyphenols can reduce Ag^+^ and Au^+^ ions to Ag and Au nanoparticles, respectively. In the present research, the Al^3+^-polyphenol complex was synthesized via the green method where Al^3+^ ions interacted with polyphenols from black tea leaf extract solution. Besides polyphenols, the other constituent of tea leaves is caffeine, as confirmed by Zielinski et al. [14]. Coordination compounds can also be recognized as coordination complexes, complex compounds, or just complexes. They are characterized by coordinate bonds formed between the ligands as electron-pair donors and the metal atom or ions as electron-pair acceptors [15]. To the best of our information, the combination of the metal complexes and polymers can be useful in the creation of polymer composites with a controlled optical bandgap. Over the past two decades, there has been considerable advancement in organic-inorganic composite materials, which tends to be a significant and undoubtedly highly fascinating research area. Physicists and chemists have proposed a wide variety of architectures with greater or lesser complexity of empirical implementation to overcome the complexity inherent in the interaction of materials with limited compatibility with one another [16].

This work aims to produce polymer composite with a small energy bandgap. In accordance with the good optical characteristics, a green technique might create polymer composites with such small *E_g_*. The improvements of optical properties through the addition of metal ion-complex (for instance, Al^3+^-polyphenol complex) into polymers have not yet been addressed. Consequently, the results of this study can be regarded as a narrative advance in polymer composites. In this study, the optical dielectric function was used precisely to recognize the sorts of optical transitions between the valence band and the conduction band experimentally. The findings indicate that some issues, such as lifetime, price, and flexibility, which limit the applicability of conjugated polymers, can be described by using small bandgap PVA together with excellent film-forming ability in order to satisfy price-performance relationships.

## 2. Materials and Methods

### 2.1. Materials

PVA powder (M_W_ from 85,000 to 124,000) and aluminum chloride hexahydrate (AlCl_3_·6H_2_O) [MW = 241.43 g/mol] were supplied by Sigma-Aldrich (Kuala Lumpur, Malaysia). The black tea leaves were purchased from the local market.

### 2.2. Sample Preparation

The extraction of tea leaves involves the use of distilled water (DW). The process is as follows: 50 g of black tea leaf was put in 250 mL DW at nearly 90 °C in the absence of sunlight. After standing for 10 min, the resulting extract solution was filtered through (Whatman paper 41, cat. No. 1441), having a pore radius of 20 µm in order to remove the residues thoroughly. In a separate flask, 10 g of aluminum chloride hexahydrate (AlCl_3_·6H_2_O) was dissolved in 200 mL of DW. The Al^3+^-polyphenol complex was then fabricated by pouring the AlCl_3_·6H_2_O solution into the extract tea leaf solution at 80 °C and stirred for 10 min. The color changes of the extract solution from dark to green and a cloudy precipitate formation proved the complexation between Al^3+^ ions and polyphenols. The resulting complex solution was left to cool down to ambient temperature. After multiple washes of the Al^3+^-polyphenol complexes with DW, these complex products were then dispersed in 100 mL of DW. The same methodology has been used to make Al^3+^-polyphenol complexes as described in the Materials and Methods part of reference [16]. The solution cast method was applied to fabricate the composite samples consisting of PVA doped with Al^3+^-polyphenol complex. First, a PVA solution was prepared by inserting 1 g of PVA to 40 mL of DW, followed by stirring for 1 hr at around 80 °C and finally cooling down to room temperature. Different volumes of 0–30 mL of the complex solution in steps of 15 mL were added to the homogeneous PVA solution. The obtained solutions were left under stirring for around 50 min. The samples were denoted as PVALMC0, PVALMC1, and PVALMC2, corresponding to 0 mL, 30 mL, and 60 mL of the added complex solution. The mixture contents were poured into Petri dishes to cast the fabricated films, and subsequently dried at room temperature. Before characterizations, the samples were further dried by using blue silica gel desiccant. The thickness of pure PVA and composite films was measured to be in the range of 0.012–0.015 cm.

### 2.3. X-ray Diffraction and FTIR Measurements

X-ray diffraction (XRD) patterns were measured on a Bruker AXS diffractometer (Billerica, MA, USA) operating at 45-mA current and 40-kV voltage at room temperature. The composite films were inspected in the wavenumber region range from 400 to 4000 cm^−1^ with a spectral resolution of 2 cm^−1^, using Nicolet iS10 Fourier-transform infrared spectroscopy (FTIR) spectrophotometer (Perkin Elmer, Melville, NY, USA).

### 2.4. UV-Vis Spectroscopy

Ultraviolet-visible (UV-vis) absorption spectra of the prepared films were recorded using a Jasco V-570 UV-Vis-near-infrared (UV-vis-NIR) spectrophotometer (Waltham, MA, USA).

## 3. Results and Discussion

### 3.1. UV-Vis and FTIR Study of Al^3+^-Metal Complex

**Figure 1:**
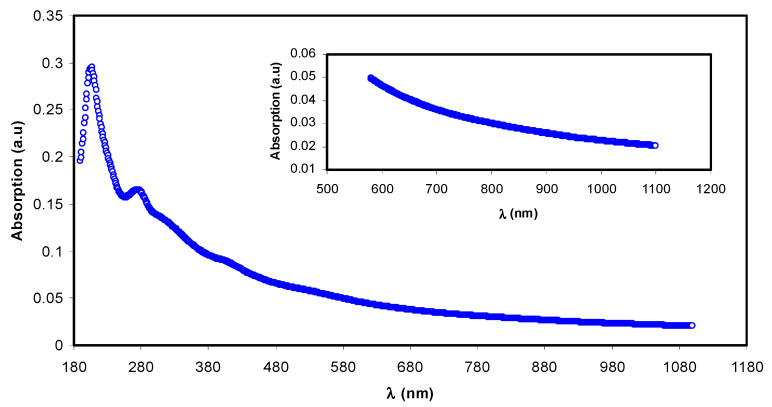
Ultraviolet-visible (UV-vis) absorption spectrum for Al^3+^-metal complex.

The absorption spectrum of the complex colloidal suspension (i.e., Al^3+^-polyphenol) is depicted in Figure 1. It is found to be comparable to the absorption spectra of semiconductors and organometallic-based materials [17,18]. The absorption spectrum notably covers the whole visible range. The inset of Figure 1 points to the Al^3+^-polyphenol complex exhibiting absorption even at high wavelength ranges to near-infrared. The present UV-vis data are like the ones demonstrated by other researchers for metal-polyphenol complexes prepared by green methods.

The absorbance band appeared from 200 to 350 nm, corresponding to the electronic transition of n–π* of catechins, methylxanthines, and caffeine. The band absorbance of C=O chromophore in caffeine appears at ~278 nm [19,20,21]. The metallic with sizes in nano range have to show a surface plasmon resonance (SPR) absorption band in UV-visible range [22]. However, the absence of this band in the present Al^3+^-polyphenol complex indicates that the polyphenols capping prevented the occurrence of metal characteristics of this complex system on particle surfaces. In previous research, chitosan-based polymer electrolytes exhibited SPR band in the range of 500 to 800 nm owing to the Cu nanoparticles [23].

**Figure 2:**
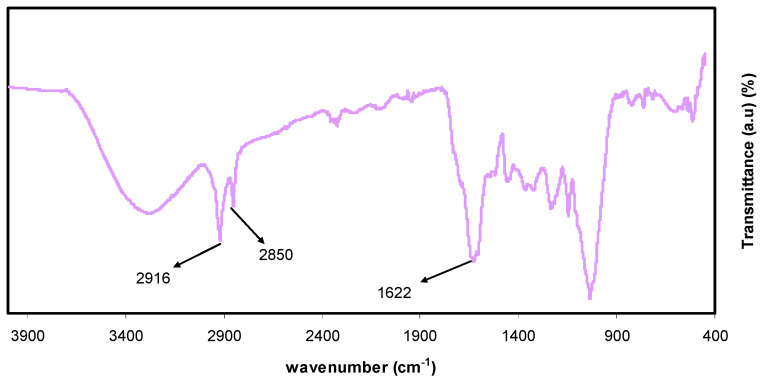
Fourier-transform infrared spectroscopy (FTIR) band of black tea leaf.

**Figure 3:**
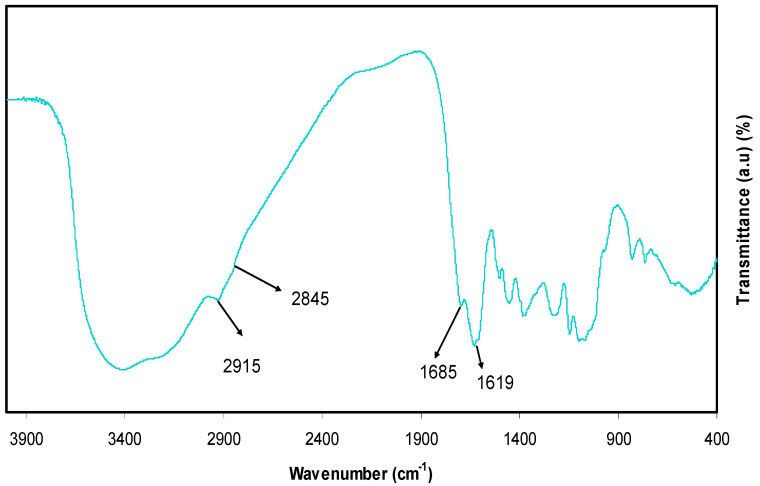
FTIR band of Al^3+^ complex.

For detecting interactions among atoms or ions in polymer electrolyte or composite materials, the FTIR method of spectroscopy is crucial. Due to these interactions, polymer electrolyte vibration modes may be altered [16]. Chemical components with exacting frequency absorbance of functional groups can be successfully analyzed via infrared (IR) spectroscopy. The more complex the structure is, the greater the absorption bands and complex spectra [24]. The FTIR spectra for the extracted black tea and colloidal Al^3+^-complex are shown in Figure 2 and Figure 3, respectively. Intense broadband centered at 3401 cm^−1^ is distinctly attributed to the N-H and O-H stretching modes of polyphenols [25,26]. Earlier studies affirmed that both O-H stretching in alcohols, phenols, and carboxylic acids and N-H stretching in (primary and secondary) amines and amides were shown up with the broadband in the range of 3410–3371 cm^−1^ [27]. In addition, C=C stretching band in the aromatic ring and C=O stretching band in polyphenols occurred at 1623 cm^−1^ [26,28]. The peaks that appeared in the range of 2920–2850 cm^−1^ have been attributed to C-H stretching vibration of aliphatic groups and carboxylic acids [26,27], while a band at 1029 cm^−1^ has been attributed to C-O stretching in amino acids [26,28].

Furthermore, the bands between 1750–1620 cm^−1^ have been ascribed to the C=O vibration of bonded conjugated aldehydes, ketones, quinines, and esters [27]. As previously reported in different researches, typical bands seen at 3388, 1636, and 1039 cm^−1^ in the analysis of tea extracts containing polyphenols have been referred to O-H/N-H stretching, C=C stretching, and C-O-C stretching vibrations, respectively [26,28,29,30]. Hence, the current study is mainly organized to show that aluminum colloidal imputed to organometallics can be virtually established by the FTIR technique. From a chemistry and physics point of view, due to the high content of polyphenols and conjugated double bonds in tea extract solution, as demonstrated by FTIR analysis, it is easy to understand the formation of organometallic compounds via green remediation, as well as the interaction of these constituents with Al-salt as a strategy of capturing Al^3+^ ions. Additionally, the formation of organometallic materials like the one between Al^3+^ ions and polyphenols is evidenced by the appearance of green solution and colloidal suspension at the top and bottom of the beaker, respectively. The optical absorption behavior of organometallic colloidal suspension will be discussed later. Normally, an intense absorption is displayed by organometallic compounds in the ranges of visible light.

**Figure 4:**
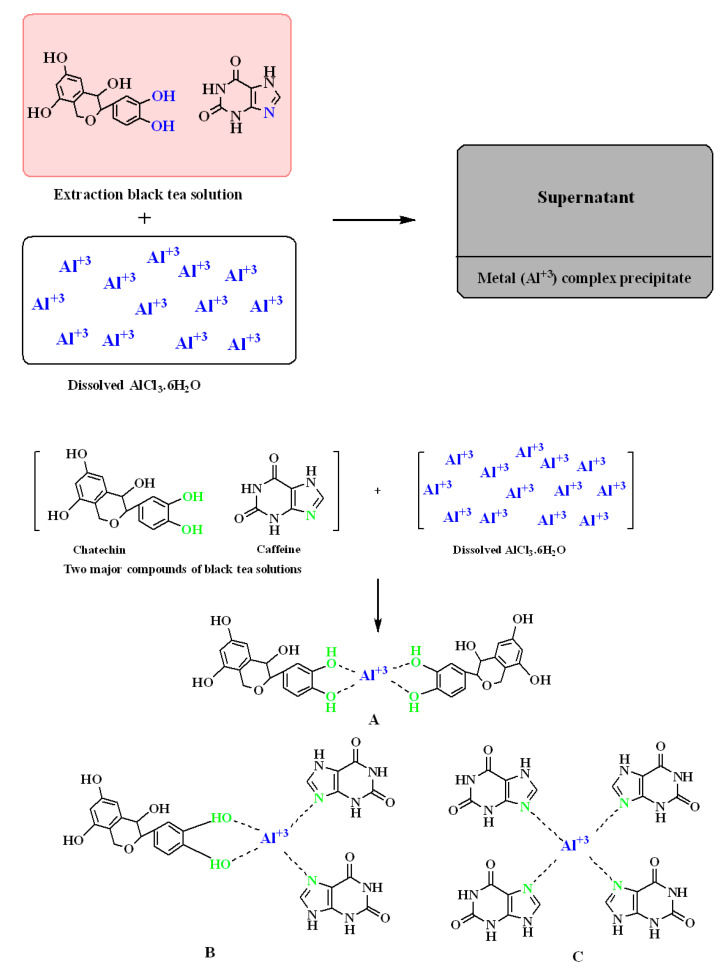
The proposed structures for the formation of (**A**) Al^3+^-polyphenol complex, (**B**) Al^3+^-caffeine complex and (**C**) complex form involving both polyphenol and caffeine components

Following earlier studies and current FTIR analysis, the complexation of Al^3+^ ions with polyphenols is shown in Figure 4. Based on the knowledge of chemistry, some complexes may be developed between Al^3+^ ions and polyphenols’ constituents. As reported, caffeine and polyphenols can interact with metal ions [31,32,33]. Figure 4 represents three different forms of expected complexes. The suggested forms of Al^3+^-polyphenol complex (Figure 4A), on the first hand, and of Al^3+^-caffeine complex (Figure 4B), on the other hand, indicate that Al^3+^ ions can form complexes with polyphenols and caffeine, respectively. In Figure 4C, one more complex form involving both of these components is suggested. However, the researchers utilized the electron paramagnetic resonance EPR routine to learn the complex formation of metal ions with polyphenols of black tea extract solution [31]. In this research, the FTIR technique was used in such studies.

### 3.2. FTIR Analysis for PVA/Al^+3^-Complex Hybrids

**Figure 5:**
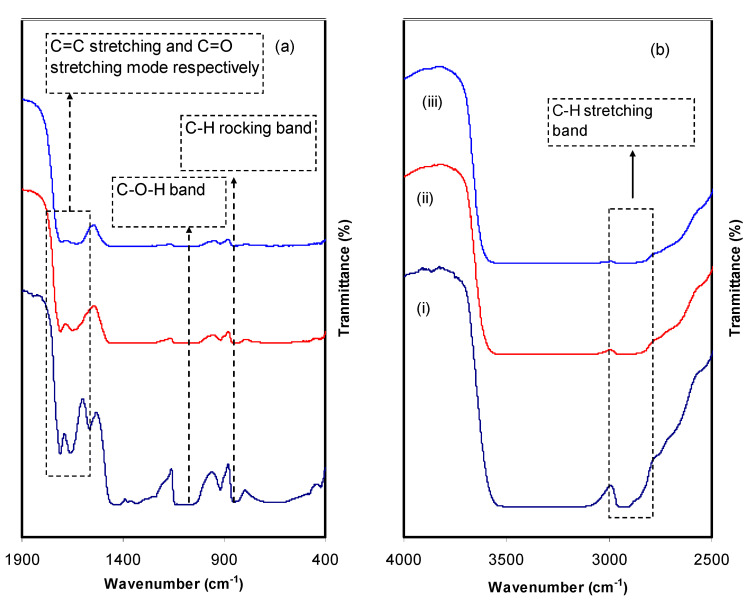
FTIR spectra of (i) PVALMC0, (ii) PVALMC1, and (iii) PVALMC2 in the region (**a**) 400–1900 cm^−1^ and (**b**) 2500–4000 cm^−1^.

FTIR methodology is a narrative advance to perceive the interaction that occurs among polymers and dopants. Such interactions can alter the vibrational modes of the polymer systems [34]. Figure 5 depicts the FTIR spectra of pure PVA (i.e., PVALMC0) and PVA doped with organometallics (i.e., PVALMC1 and PVALMC2). The absorption peak at 824 cm^−1^ is caused by the C-H rocking of pure PVA [34]. This peak is shifted, and its intensity dropped for the PVA hybrid samples while it almost disappears after adding 60 mL of dopant material. CH_2_ wagging has been recognized as the basis for the pure PVA absorption peak at 1410 cm^−1^, while C-OH plane bending has been linked to the pure PVA absorption peak at 1316 cm^−1^ [35]. Thus, the composite films are characterized by the shifting of peaks and a significant drop in peak intensity. Meanwhile, the broad and intense absorption peak at 3340 cm^−1^ can be assigned to the O-H stretching vibration of hydroxyl groups [36]. The significant intensity of this peak might be owing to the well-built intra- and intermolecular H-bonding [34]. Furthermore, this peak is shifted and exhibits much lower intensity in the doped samples. In pure PVA, the peak at 1644 cm^−1^ belongs to C=O stretching of the acetate group, which is shifted to the lower wavenumber in the doped samples [35]. C-H asymmetric stretching vibration provides an absorption band at 2908 cm^−1^ [36,37], which is shifted and diminished considerably in PVA composites. In addition, a featured peak for –C–O– stretching vibration in pure PVA appears at 1076 cm^−1^ [38], which is altered and loses some of its intensity in the composite films. Two explanations for declaring the peak intensity fluctuation and falling include the interaction among PVA-OH groups and metal complex and the adsorption of metal complex colloidal on the host polymer’s functional groups. Consequently, since adsorption causes an increase in molecular weight M_W_, a decline in the functional groups’ vibrational intensity can be observed [39].

### 3.3. XRD Study of PVA/Al^+3^-Complex Hybrids

**Figure 6:**
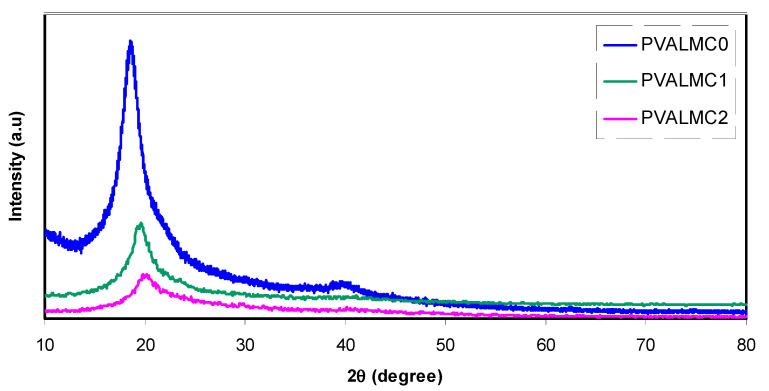
X-ray diffraction (XRD) pattern for the PVALMC0, PVALMC1, and PVALMC2 samples.

Studying the X-ray diffraction pattern of polymer nanocomposites is a reliable and straightforward technique to analyze the crystal structure. It has been shown that the addition of fillers enhanced the amorphous phase of polymers through the disruption of hydrogen bonding and resulted in the formation of an amorphous structure. Figure 6 shows a sharp diffraction peak for pure PVA at 2θ = 19°, which corresponds to the crystalline structure and agrees with the literature [16,40,41]. Despite a decrease in the intensity of these peaks, they remained in the doped PVA spectra. Significantly, the peak at 2θ = 40° almost disappeared. This is mainly attributed to the weakening of the intermolecular forces inside the PVA polymer, which reflects an almost increase in amorphous phases.

On the other hand, the peak at 2θ = 20° was broadened. Growing the amorphous phase explains both of these width increments and intensity decrement of the peak at 2θ = 20° [42,43,44]. Our previous investigation revealed that incorporating the Zn^2+^-polyphenol complex into the chitosan polymer electrolyte significantly enhanced the amorphous phase [45].

Presenting both amorphous and crystalline regions in XRD data of PVA composite films may indicate the semi-crystalline nature of the matrix. However, the remaining main peak positions of PVA in its position suggest that some original crystal structure of the polymer was not affected by the filler.

### 3.4. Optical Properties

#### 3.4.1. Absorbance and Absorption Edge Study

**Figure 7:**
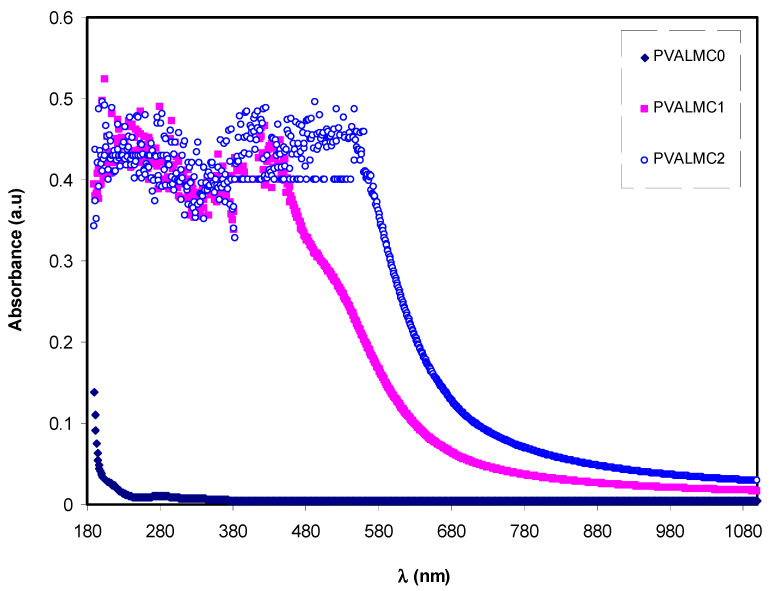
Absorption spectra for the PVALMC0, PVALMC1, and PVALMC2 samples.

Figure 7 illustrates the absorption spectra of pure PVA polymer and their composites. The absorption spectrum of composite alloys covers almost all the significant parts of solar radiation. Most organometallics commonly display prominent optical absorption and emission in the 600–700 nm regions [46]. This may be interpreted based on overlap formation between the orbitals with the assistance of ligands. Thus, electrons can carry energy all over the structure and provide the absorption spectra [47]. Thus, it is implicit that it is possible to expect some phenomena from the optical absorption spectra, including reducing the optical bandgap. This is related to the fact that optical constants include valuable information for technological applications. The optical properties of polymers are essential for optical applications because optical properties are directly related to their structural and electronic properties [46]. It is observed that the optical absorption edge is not sharply apparent, which indicates the amorphous nature of the samples [48].

The absorbance of pure PVA in the (240–270 nm) region is in agreement with previous studies and is ascribed to the π–π* electronic transition in the aromatic ring group, which has mainly occurred due to forbidden transitions in the excited states of the polymer. The absorption between 270 nm and 375 nm was associated with the *p*–p* electronic transition in –C=C– bonds [49].

**Figure 8:**
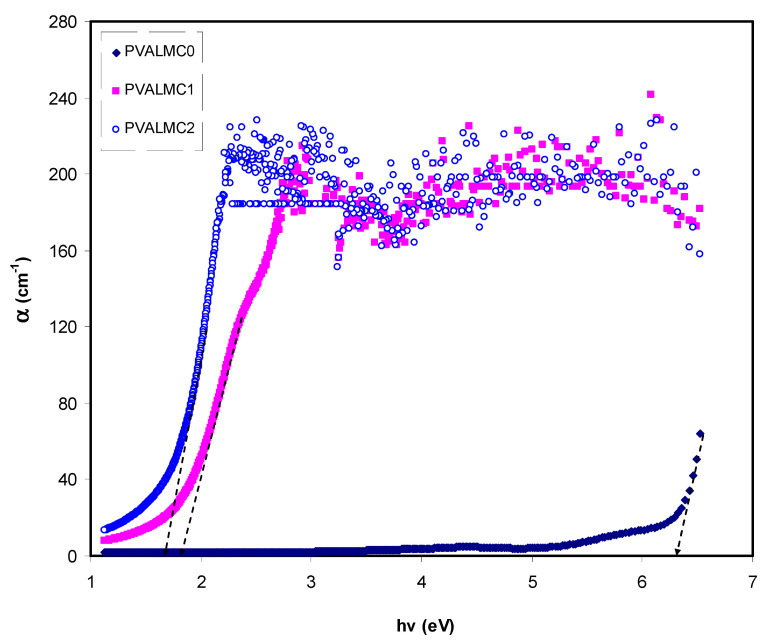
The plot of α versus hν for the PVALMC0, PVALMC1, and PVALMC2 samples.

Figure 8 demonstrates the absorption coefficient (α) of clean PVA polymer and their hybrids. The following equation can be employed to calculate the α of the prepared films at various *λ* from the absorption patterns [39]:

(1) $$\alpha \;=\;\frac{\ln(1/T)}{t}=(2.303)\times \left[\frac{A}{t}\right]$$

Here *t* is the thickness of the films. Investigations of optical absorption of polymer/metal complex hybrids, particularly the absorption edge, have been shown to be a valuable tool to elucidate these materials’ electronic structures. Characterization of indirect and direct transition happening in the bandgap of polymer/metal complex hybrids is feasible through optical absorption spectra [50]. Meaningful information on the optical band gap is achieved when the fundamental absorption edge (FAE) is well studied. In the absorption process, an absorption edge represents the action of exciting an electron by a photon from a lower to a higher energy state [51]. The broad shift of FAE to lower photon energy for the hybrid films than pure PVA, as depicted in Figure 8, is evidence that metal complexes are essential for doping functional polymers. The FAE values are presented in Table 2.

#### 3.4.2. Refractive Index and Localized Density of State (N/m*) Study

**Figure 9:**
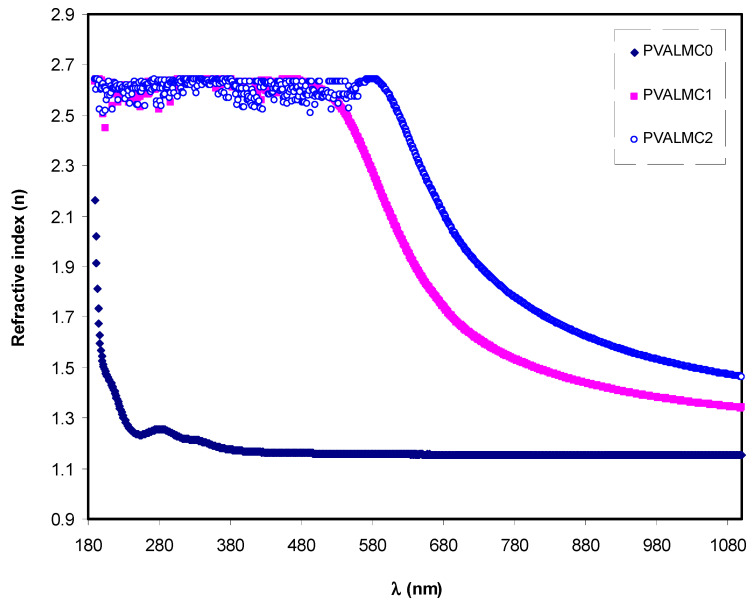
Refractive index (*n*) against wavelength (*λ*) for the PVALMC0, PVALMC1, and PVALMC2 samples.

Figure 9 illustrates the indices of refraction (*n*) of neat PVA and their hybrids. Clarified dispersion in the *n* pattern can be observed for the hybrid films. From the optical materials point of view, the detailed knowledge of the wavelength-dependent complex index of refraction, n, is vital for the optical system’s proposal and routine [52]. One of the significant characteristics of material includes the refractive index since it is highly connected to both the electronic polarizability of ions and the internal field. Therefore, its assessment for optical materials is highly important for applications in integrated optic devices like switches, fififilters, and modulators. The refractive index is regarded as a critical parameter in designing those devices. [53]. Besides its function in optical devices, the variations in *n* are crucial for scheming the optical characteristics of polymers [54]. The values of refractive indices were calculated from reflectance (*R*) and extinction coefficient (*K*) by using Kramer’s–Kronig relations:

(2) $$n=\frac{1+R}{1-R}+\sqrt{\frac{4R}{{\left(1-R\right)}^{2}}-K^{2}}$$

where *K* = *λα*/4π*t*, *λ* is the wavelength of incident light and *t* is the thickness of the prepared films. The results shown in Figure 9 suggest that the films’ refractive index could be increased by incorporating Al-complex. It also demonstrates that all the tested specimens’ refractive indices were maximum in the UV region below 300 nm. After that, they started to decrease with the increase of wavelength. At long-wavelength (*λ*→∞), the value of *n* became constant. This is because of the resonance effect due to the specimens’ polarization by the incident light’s photons. The refraction index for pure PVA exhibited a sharp decline in the wavelength range of 300 nm then remained steady.

On the other hand, the index of refraction of PVA composites increased as Al complex concentration increased, and it demonstrated a gradual decline with the increase of wavelength. This is possibly due to the enhancement of packing density and PVA polarization by adding the Al^3+^-complex [55]. Figure 9 indicates that the Al complex filler’s insertion into the PVA polymer matrix altered the *n* of the composite films and increased the value from 2.2 to about 2.7. This boost in the value of the static refractive index by nanofiller addition is consistent with the previous studies. It is likely ascribed to the enhancement of bond strength and dipole strength due to the creation of space charges in the presence of the Al complex [56].

The basic optical transition in polymer composites is mainly attributed to the variation of optical dielectric constant, which describes the feasibility of losing energy by an electron as it travels through a surface of the bulk material. It is expressed in terms of real (*ԑ_r_*) and imaginary (*ԑ_i_*) parts. The real part measures the ability of the material to slow down the speed of the electromagnetic wave. The imaginary part accounts for the efficiency of absorbing energy due to polarization. The value of *ԑ_r_* is calculated from the refractive index (*n*) of the medium (*ԑ_r_* = *n*^2^ − *k*^2^), while the value of *ԑ_i_* is derived from extinction coefficient (*k*) (*ԑ_i_* = 2*nk*).

**Figure 10:**
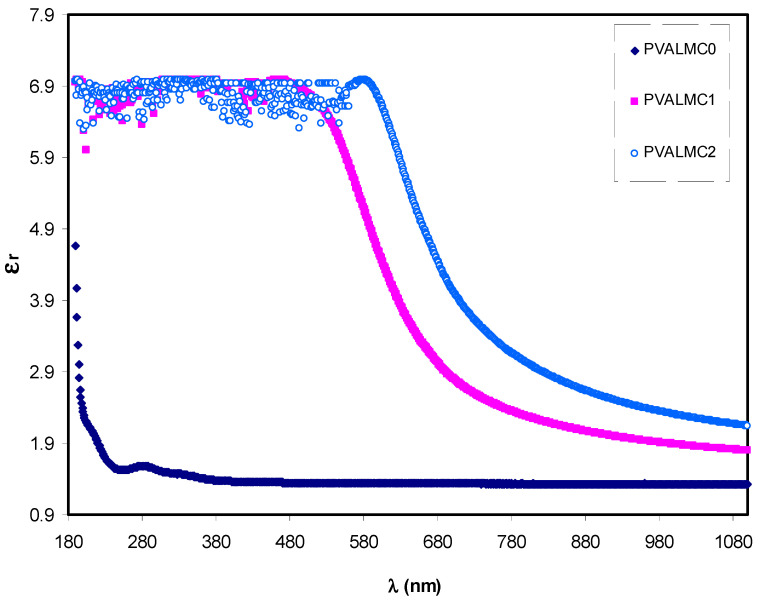
Optical dielectric constant (*ԑ*_r_) against wavelength (*λ*) for the PVALMC0, PVALMC1, and PVALMC2 samples.

Optical dielectric constant (*ԑ*_r_) against wavelength (*λ*) for the PVALMC0, PVALMC1, and PVALMC2 samples is shown in Figure 10. Dielectric response of the material at high frequency (short wavelengths) *ԑ*_∞_ was determined from the correlations between wavelength and refractive index according to Spitzer–Fan model [57]:

(3) $$\varepsilon_{r}=n^{2}-k^{2}=\varepsilon_{\infty }-(\frac{e^{2}}{4\pi^{2}\;C^{2}\;\varepsilon_{o}})\;\times (\frac{N}{m^{*}})\lambda^{2}$$

where *e* is the charge of an electron, *c* is the speed of light, *ԑ_o_* is the dielectric constant of free space, *N* is the concentration of charge carrier, and m* is the effective mass it is assumed to be 1.16 *m*_e_ [58,59].

**Figure 11:**
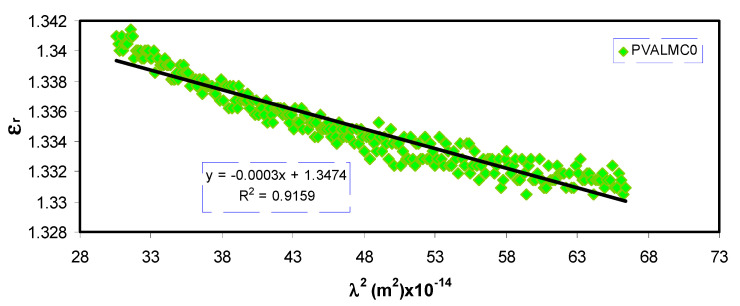

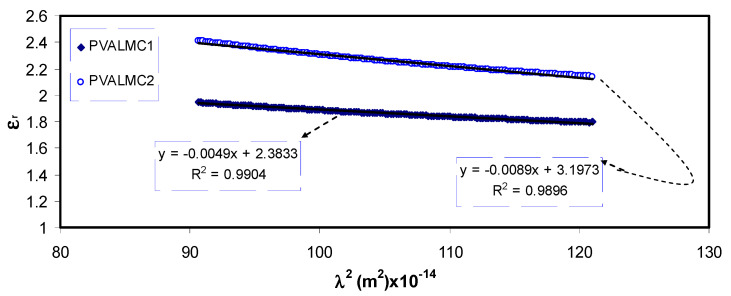
The plots of *ԑ_r_* versus *λ*^2^ for the PVALMC0, PVALMC1, and PVALMC2 samples.

Plotting the values of *ԑ*_r_ versus *λ*^2^ in the visible wavelength region yields a straight line, as shown in Figure 11. The *ԑ*_∞_ and N/m* values were determined from the slope and intercept of the line with the y-axis, respectively, using the parameters shown in Table 1. The values of *ԑ*_∞_, N/m*, and N estimated from Equation (3) are summarized in Table 2.

From Table 2, it can be noticed that as filler concentration increased, the values of charge carriers/m* for pure PVA film raised by up to 20 times from 3.68 * 10^55^ to 109 * 10^55^ atoms/m^3^, and the value of *ԑ*_∞_ increased from 1.4 to 3.6, indicating that the increase of free charge carriers had vigorously participated in the polarization process. The values estimated for localized density of states (N/m*) in the present work are comparable with those reported in the literature by other researchers using Equation (3) [60,61].

#### 3.4.3. Optical Dielectric Losses and Tauc’s Model

The quantum mechanics (QM), especially the complex dielectric function (CDF) assessment, need to be considered to describe this structure-property relationship accurately. This is because the CDF expresses the electron density of a material to an external electromagnetic fififield [62]. It is hard to forecast the type of electron transition from the Tauc equation because exponents should be studied, as can be seen in later sections [63]. Previous theoretical studies confirmed the existence of a considerable connection between the CDF (ε^*^= *ε*_1_ − *i ε*_2_) and the band structure of insulating and semiconductor materials. In fact, ε* represents the nature of the medium in response to the propagation of the electromagnetic wave through it. The imaginary part *ε*_2_ represents the real transitions between the occupied $\Psi_{k}^{\nu }$ and unoccupied $\Psi_{k}^{c}$ wave functions (electronic states), and is given by [64]:

(4) $$\varepsilon_{2}=\frac{4\pi^{2}e^{2}}{m^{2}\omega^{2}V}\sum_{\nu ,c,k}\left|\langle \Psi_{k}^{\nu }|\vec{p_{i}}|\Psi_{k}^{c}\rangle \right|\delta \left(E_{\Psi_{k}^{c}}-E_{\Psi_{k}^{\nu }}-\hbar \omega \right)$$

**Figure 12:**
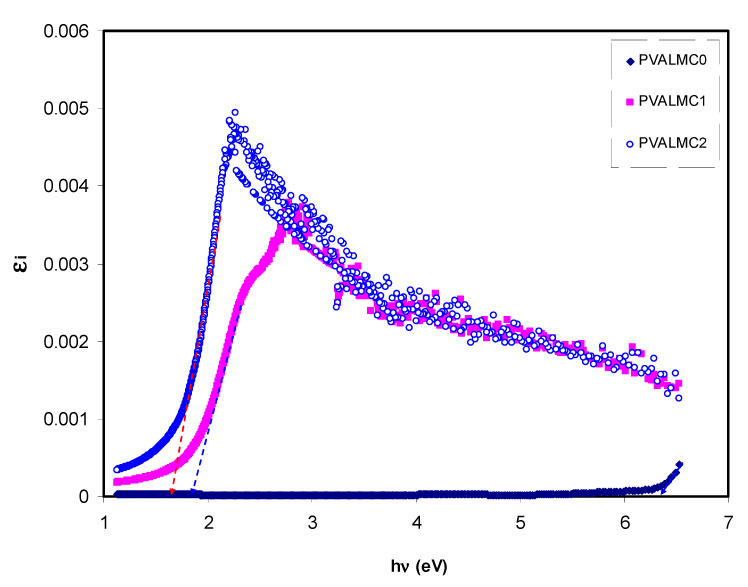
Imaginary part of dielectric constant (*ε_i_*) against hν for the PVALMC0, PVALMC1, and PVALMC2 samples.

Thus, from Equation (4), there is an apparent correlation between *ε*_2_ or *ε_i_* and the band structure $\left(E_{\Psi_{k}^{c}}-E_{\Psi_{k}^{\nu }}\right)$ from the QM point of view. The CDF being interconnected to other assessable optical parameters (*n* and extinction coefficient) can be evaluated by simple equations. Figure 12 represents the plot of *ε_i_* versus *hν* for clean PVA and composite films. Clear peaks for all the films can be detected. Prior investigations show that the peaks appearing in the *ε*_2_ part of the dielectric function are directly related to inter-band transitions [65,66]. Thus, the intercept of linear parts below the peaks (Figure 12) on the hν axis can be regarded as the valid bandgap.

Solids’ optical characteristics can be primarily explained by the CDF being linked to other detectable optical quantities using simple equations [67]. Previous studies emphasized that optical dielectric functions (*ε_r_* and *ε_i_*) are related to the density of localized electronic states within the forbidden gap of the composite films [60,61,67]. Using the *ε_i_* parameter with the help of (N/m*) values, various important other parameters such as relaxation time (*τ*), plasma frequency (*ω*_p_), and electrical resistivity (*ρ*) can easily be estimated using the Drude free electron model:

(5) $$\varepsilon_{i}=J\;(\frac{1}{\tau })\lambda^{3},\;\;\;J=\frac{e^{2}}{8\pi^{3}c^{3}\varepsilon_{o}}\frac{N}{m*}$$

**Figure 13:**
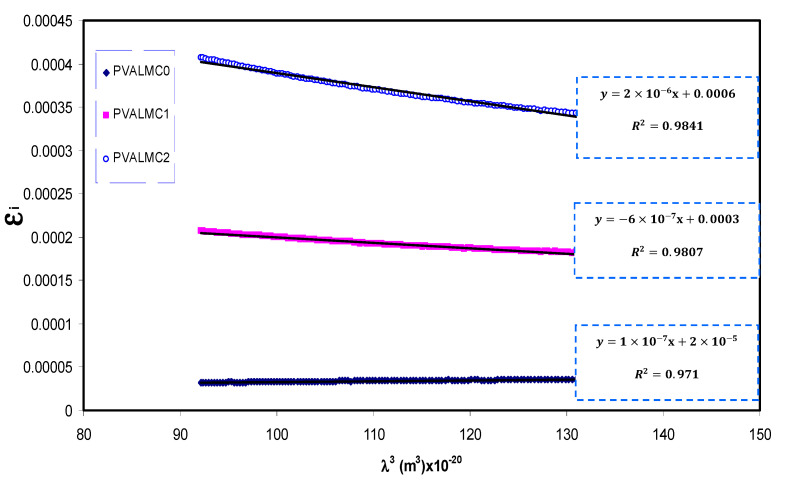
*ε_i_* against *λ*^3^ for the PVALMC0, PVALMC1, and PVALMC2 samples.

Figure 13 shows a variation of *ε_i_* with *λ*^3^ for pure PVA films at different Al metal complex loadings in the region where a linear behavior was achieved. Using the N/m* values obtained from Equation (5) and the slope of *ε_i_* versus *λ*^3^, the values of relaxation time (*τ*) were calculated. In addition, the optical mobility (*µ_opt_*), electrical resistivity (*ρ_opt_*), and plasma angular frequency of the electron were computed from the following relations [68]:

(6) $$\mu_{opt}=\frac{e\tau }{m^{*}}$$

(7) $$\rho_{opt}=\frac{1}{e\mu_{opt}N_{c}}$$

(8) $$w_{p}=\frac{e^{2}N}{\varepsilon_{o}m*}$$

The calculated values of *τ* and *ω_p_* are also presented in Table 3. It can be seen from the table that the values of relaxation time (*τ*), optical mobility (*µ_opt_*), and optical resistivity (*ρ_opt_*) of pure PVA were dropped with the addition of the Al^+3^ metal complex. This indicates that the nanocomposites’ relaxation answer to the incident optical electric field has occurred quicker than the unfilled polymer. The decline of *τ*, *µ_opt_*, and *ρ_opt_* are linked to the increase of *n,* and, consequently, the velocity of light decreases in the medium with a higher refractive index. The addition of the Al^+3^ metal complex also caused an intensification of plasma frequency (*ω_p_*) of the electron by up to 20-fold, from 0.32 × 10^15^ to 1.77 × 10^15^ Hz. This is in agreement with previous findings for other polymer nanocomposites, indicating that the strong local electric field was induced by the nanofillers’ dipole moment, which enhanced the material’s polarization under the incident electric field [69]. Thus, in addition to bandgap estimation from optical dielectric loss function, various optical parameters were determined, which are crucial from the materials selection viewpoints for optoelectronic applications.

**Figure 14:**
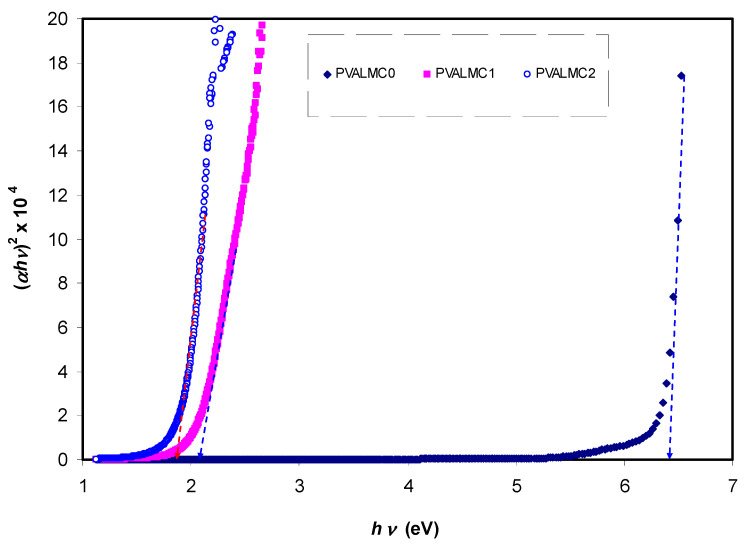
Plot of (*ahv*)^2^ against hν for the PVALMC0, PVALMC1, and PVALMC2 samples.

**Figure 15:**
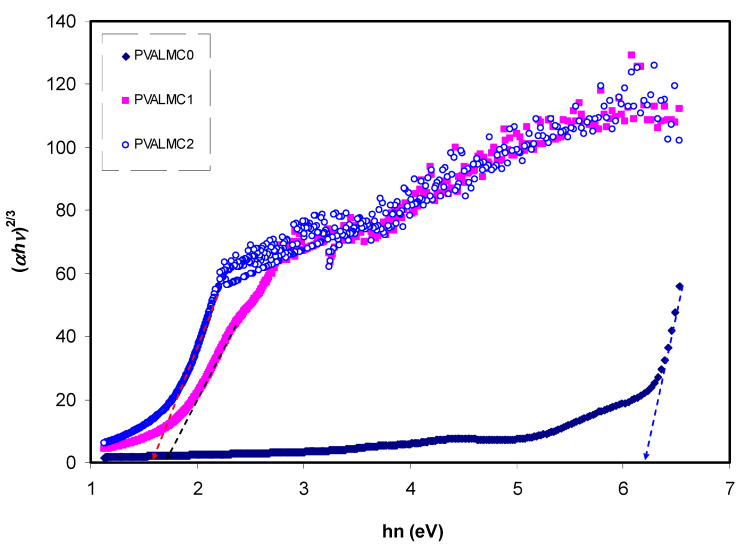
Plot of (*ahv*)^2/3^ against hν for the PVALMC0, PVALMC1, and PVALMC2 samples.

The fundamental absorption refers to the band-to-band transitions, which are treated with exact selection rules, and it declares itself by a speedy boost in the fundamental absorption region [50,70]. Based on the band structure, there are several types of transitions [70]. In amorphous semiconductors which have indirect transitions, no electronic momentum conservation is available when the transition occurs from the valence band to the conduction band [47]. The *α* for direct bandgap material is expressed by the equation below [63]:

(9) $$\alpha =\frac{A}{h\nu }{\left(h\nu -E_{g}\right)}^{\gamma }$$

where *hυ* is photon energy, *A* is a constant, and *E_g_* is optical energy gap. The values of *γ* = 1/2, 3/2, 2, and 3 representing direct allowed, direct forbidden, indirect allowed, and indirect forbidden transitions, respectively. The intercept of linear parts of Figure 14 on the photon energy axis can be employed to evaluate the band gap value. It is well reported that the success of optoelectronic tools is associated mainly with the current enhancement in material superiority. Knowledge of band gaps is crucial for understanding a semiconductor’s electrical properties and is therefore of great practical interest [70]. The bandgap values for direct allowed transition (*γ* = 1/2) and direct forbidden transition (*γ* = 3/2) are presented in Table 4. Since different orbitals of the metal complexes and the ligands contribute to the band edges in amorphous materials, it is hard to envisage whether the transition type is direct or indirect [71]. Thus, from the fundamental absorption equation (i.e., Equation (3)), many figures can be drawn in reference to the values of *γ*. However, it is hard to specify the type of electron transition from Equation (3) alone. To identify the responsible type of electron transition accurately, the Tauc’s model results should be compared to bandgap values received from *ε_i_* spectra (Figure 12). From the comparison of Figure 14 and Figure 15 to Figure 12 (see Table 4), it can be deduced that the type of electronic transition in pure PVA is direct allowed, and in composite samples it is forbidden direct allowed transition. Thus, an optical dielectric function is an effective tool to study the band structure of solids. The source and the distinct peaks in the dielectric functions of carbon-based materials are caused by interbond transitions [66].

The bandgap variation reported in the present work for PVA impregnated with Al^3+^-complex is of great importance compared to those reported for PVA incorporated with various ceramic fillers and nanoparticles, as depicted in Table 5.

#### 3.4.4. Wemple-DiDomenico (WD) Model

One of the most essential properties of the optical material is the refractive index and its dispersion behavior. The refractive index dispersion is crucial in optical communication and in designing devices for spectral dispersion [81]. The single oscillator model presented by WD can be applied to explore the refractive index dispersion in the normal region [82]. The exploration is done by introducing a dispersion energy parameter (*E_d_*) as a gauge of the force of inters band optical transition. This *E_d_* parameter combines both the coordination number and the charge allocation in each unit cell and is firmly interconnected to the chemical bonding [83]. However, a single oscillator parameter (*E_o_*) is proportional to the energy of the oscillator. The refractive index and the photon energy below the interband absorption edge can be related to each other through the following semi-empirical relation:

(10) $$n^{2\;}-1=\frac{Ed\;Eo}{\left[Eo^{2}-{\left(hv\right)}^{2}\right]}$$

**Figure 16:**
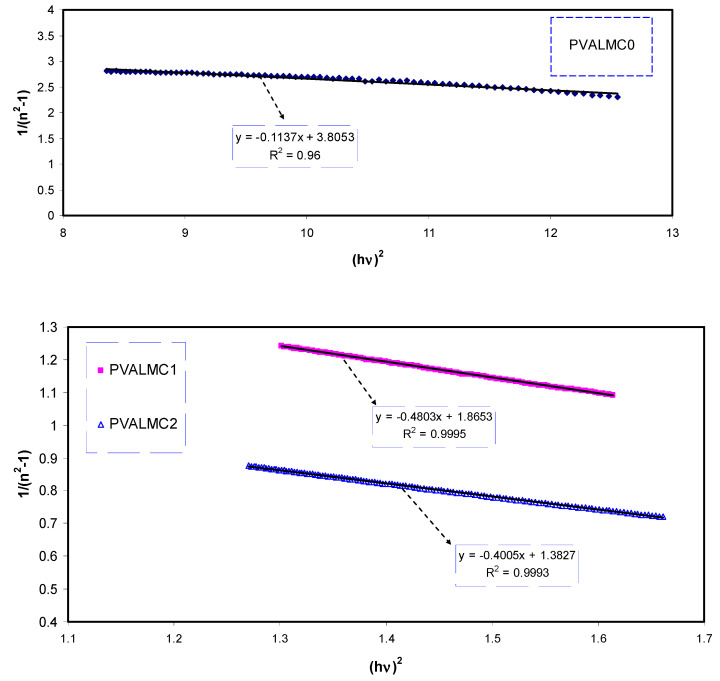
Variation of (*n*^2^ − 1)^1/2^ versus photon energy (*hν*)^2^ for the PVALMC0, PVALMC1, and PVALMC2 samples.

As depicted in Figure 16, the data on the plots of 1/(*n*^2^ − 1) against (*hυ*)^2^ were fitted with linear regression lines to obtain the values of *E_d_* and *E_o_* from the intercept and slope, respectively. The calculated values of the *E_o_* and *E_d_* are given in Table 6. An increase in *E_d_* and a decrease in *E_o_* values with increasing the concentration of the Al^3+^ complex solution were observed. There is a relation of *E_o_* with the optical band gap (*E_g_*) [84]. For the present films, empirically, the *E_o_* values are equal to the direct *E_g_* (i.e., *E_o_ ≈ E_g_*), as shown in Table 4.

## 4. Conclusions

In conclusion, the PVA composite films with various amounts of the Al^3+^-metal complex were made using a solution casting technique. The UV-visible spectroscopy was employed to analyze the influence of dispersion of the Al^3+^-metal complex on the optical properties of the PVA. Studies of UV-visible spectroscopy and FTIR showed that the complexation of organometallic and PVA host matrix has occurred. The XRD analysis revealed that the black tea contained sufficiently functional groups and double bonds that were conjugated. An increase in width and a decrease in the intensity of the XRD patterns showed the increment of the amorphous phase inside the matrix. The FTIR technique demonstrated the band shifts and intensity drops in the PVA doped polymers. To obtain the optical bandgap, Tauc’s equations were used to specify the type of electronic transition. The derived bandgap for the composite films was comparatively small and close to those achieved for inorganic semiconductor-based materials. It was observed that the optical bandgap decreased significantly.

In contrast, optical parameters such as (*ϵ_r_* and *ϵ_i_*) and (*n*) were considerably enhanced with the increased concentration of the Al^3+^-metal complex in the PVA matrix. Accordingly, to evaluate the dispersion parameters of (*E_o_*), (*E_d_*), and (*n_o_*) of the prepared composites, the theoretical Wemple-DiDomenico single oscillator model has been used. The refractive index was quite well-tuned once organometallic was added to the PVA polymer. The optical dielectric loss was precisely analyzed in order to measure the optical bandgap. Thus, a detailed analysis of these prepared composites’ optical parameters has a broad potential for extensive use in optoelectronic devices due to their flexibility and optical bandgap.

## Figures and Tables

**Table 1:** Various physical parameters were used for the calculation of localized density of states (N/m*) for the prepared poly(vinyl alcohol) (PVA)/Al^3+^-complex hybrids.

Physical Parameters	Values
mass of an electron (*m*_e_)	9.109 × 10^−31^ Kg
charge of an electron (*e*)	1.602 × 10^−19^ coulombs
dielectric constant of free space (*ԑ_o_*)	8.85 × 10^−12^ F/m
π	3.14
speed of light (*c*)	2.99 × 10^8^ m/s
effective mass (m*)	10.566 × 10^−31^ Kg

**Table 2:** Values of optical dielectric parameters estimated for PVA containing Al^3+^- metal complex.

Film Code	N/m* × 10^55^ (m^3^/kg)	*ε*_∞_
PVALMC0	3.68	1.4
PVALMC1	60.1	2.6
PVALMC2	109	3.6

**Table 3:** Calculated values of relaxation time (*τ*), optical mobility (*µ_opt_*), optical resistivity (*ρ_opt_*), and plasma angular frequency (*ω_p_*).

Film Code	*τ* (fs)	*μ* (opt) × 10^−2^	(*Nc*) × 10^25^	*ρ* (opt)	*ω_p_* × 10^15^
PVALMC0	1.59	161	3.89	1.00 × 10^−7^	0.32631
PVALMC1	4.33	9.44	63.5	1.04 × 10^−7^	1.3188
PVALMC2	2.36	6.02	115	9.03 × 10^−8^	1.77736

**Table 4:** Bandgap values from imaginary part of dielectric constant (*ε_i_*) spectra and Tauc’s model.

Sample Code	Direct Bandgap (eV)	Indirect Bandgap (eV)	Energy gap (*E*_g_) from *ε_i_* Spectra (eV)
PVALMC0	6.4	6.2	6.39
PVALMC1	2.1	1.74	1.79
PVALMC2	1.81	1.62	1.68

**Table 5:** The bandgap variation for PVA is incorporated with various ceramic fillers and nanoparticles.

PVA Composite	Direct Bandgap (eV)	Indirect Bandgap (eV)	References
PVA:Fe	2.8	-	[72]
PVA:Al	5.2	-	[73]
PVA:Ag	-	4.78	[74]
PVA:Fe_2_O_3_	-	4.8	[75]
PVA:BaTiO_3_	5.4	4.2	[76]
PVA:SiO_2_	5.2	4.35	[77]
PVA:MnCl_2_	4.99	4.93	[78]
PVA:ZnO	5.15	-	[79]
PVA:PbO	4.33	-	[80]
PVA:Al^3+^-complex	1.81	1.62	Present work

**Table 6:** Optical bandgap from the theoretical Wemple-DiDomenico (WD) single oscillator model.

Sample Code	Dispersion Energy (*E_d_*)	Oscillator Energy (*E_o_*)	Refractive Index (*n_o_*)
PVALMC0	1.620084	6.17252	1.123596
PVALMC1	1.058334	1.968502	1.240014
PVALMC2	1.341104	1.864135+	1.311268

## Data Availability

The data presented in this study are available on request from the corresponding author.
